# Scaling red-brown hyperkeratotic papules and plaques involving the axilla

**DOI:** 10.1016/j.jdcr.2025.08.023

**Published:** 2025-08-30

**Authors:** Michael C. Elias, Christine C. Tam

**Affiliations:** a Certified Dermatologists, North Royalton, Ohio; b Department of Medicine, Southwest General Health Center, Middleburg Heights, Ohio

**Keywords:** axillary eruptions, axillary granular parakeratosis, granular parakeratosis, intertriginous eruptions

## Case description

A 65-year-old female with a history of hypertension and seizures presented with a 1-year to 2-year history of a pruritic eruption involving her axillae. The eruption was previously treated with betamethasone dipropionate lotion, resulting in temporary clearance followed by recurrence after discontinuation of treatment. She reported flares triggered by exposure to certain antiperspirants, deodorants, and the lubricating strip on her razor.

**Fig 1:**
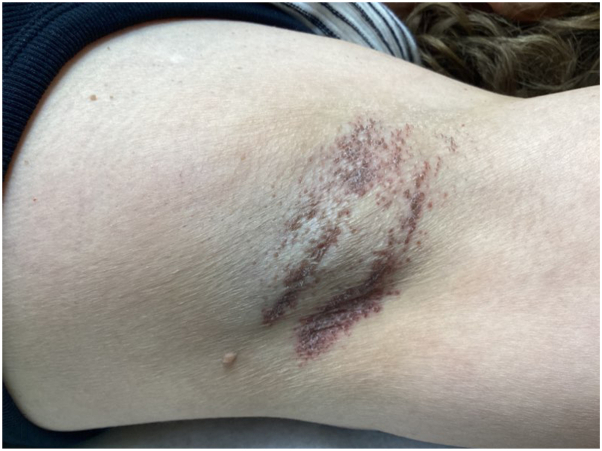
Scaling, *red-brown*, hyperkeratotic papules and plaques involving the axilla.

**Fig 2:**
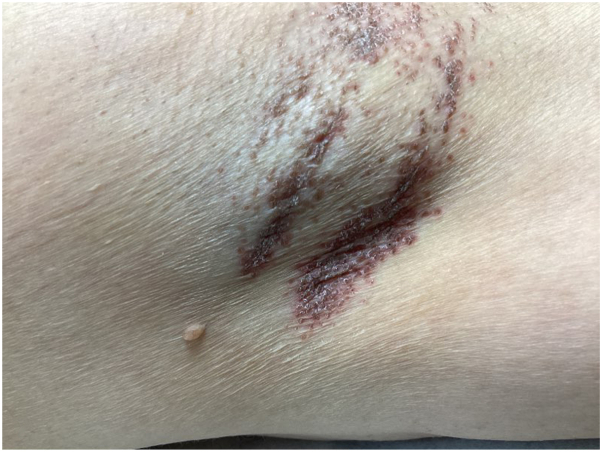
Close-up view of scaling, *red-brown*, hyperkeratotic papules and plaques involving the axilla.

**Fig 3:**
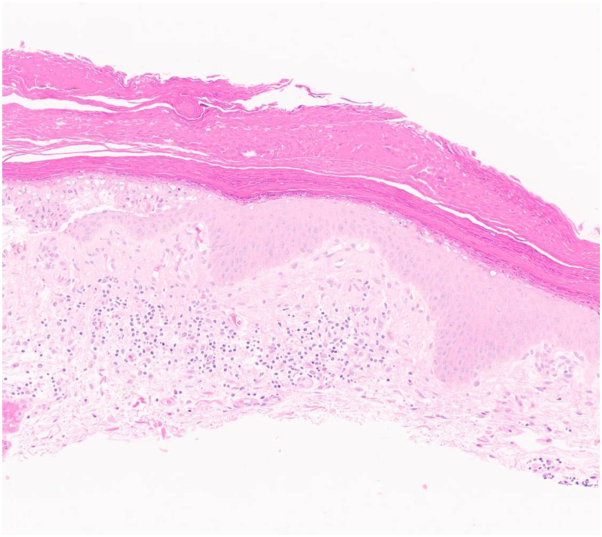
Hyperkeratosis, parakeratosis, retained keratohyalin granules within the stratum corneum, epidermal hyperplasia, and lymphocytic superficial dermal infiltrate (hematoxylin and eosin, 100× original magnification).

**Fig 4:**
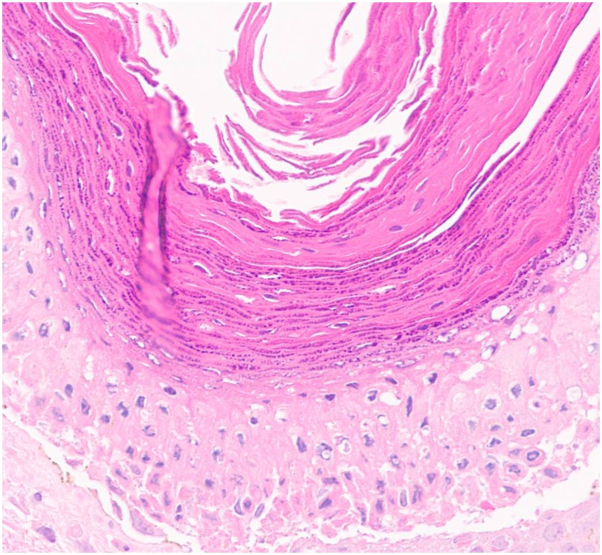
Hyperkeratosis, parakeratosis, and retained keratohyalin granules within the stratum corneum (hematoxylin and eosin, 400× original magnification).

Physical examination revealed scaling, red-brown, hyperkeratotic papules coalescing into thin plaques involving her left axilla (Figs 1 and 2). A biopsy demonstrated hyperkeratosis, parakeratosis, retained keratohyalin granules within the stratum corneum, epidermal hyperplasia, and a lymphocytic interstitial and perivascular infiltrate in the superficial dermis (Figs 3 and 4).

Her eruption cleared within 11 days of treatment with crisaborole 2% ointment applied twice daily. She was cautioned to avoid using potent topical steroids, including betamethasone dipropionate, on the axillae due to the risk of steroid atrophy.

**Question: What is your diagnosis?**

**A.** Contact dermatitis
**B.** Erythrasma
**C.** Intertrigo
**D.** Granular parakeratosis
**E.** Familial benign pemphigus

## Discussion

Granular parakeratosis is a rare, idiopathic, benign disorder typically affecting the intertriginous regions. It has been reported across all ages and is seen more commonly in women. The estimated incidence is 0.005%, although it is likely under-recognized and under-reported.^1^

The etiology remains unknown. It may be related to irritation from occlusion, friction, and topical agents such as antiperspirants, deodorants, zinc oxide, and products containing benzalkonium chloride. Cases have been linked to obesity and, in infants, occlusion from diapers.^1^^,^^2^

Granular parakeratosis typically presents as scaling, erythematous to brown patches, and hyperkeratotic papules coalescing into plaques. It usually involves the intertriginous regions, most frequently the axillae, followed by the groin, although nonintertriginous eruptions have been reported. It is often bilateral but may be unilateral as well. Eruptions are commonly pruritic and, less commonly, painful or asymptomatic.^1^

Pathology demonstrates the retention of basophilic keratohyalin granules in the stratum corneum, along with hyperkeratosis, parakeratosis, psoriasiform or papillomatous epidermal hyperplasia, and a lymphocyte-predominant interstitial or perivascular infiltrate in the superficial dermis.^1^

Response to various treatment modalities has been inconsistent.^3^ Topical medications, including corticosteroids, vitamin D analogs, keratolytic agents, retinoids, and calcineurin inhibitors; systemic treatment with oral antibiotics, isotretinoin, antifungals, and dexamethasone; and destructive modalities with cryotherapy and lasers have all had varying success. Some cases resolve spontaneously, while others abate with avoidance of precipitating factors.^1^ Recurrent and persistent cases have been reported.^3^

We report a case of granular parakeratosis that resolved with crisaborole ointment. Crisaborole ointment is a treatment that should be considered if the eruption persists despite modification of environmental factors, especially when intertriginous involvement precludes the long-term use of topical corticosteroids due to the risk of steroid-induced atrophy.

## Conflicts of interest

None disclosed.
